# Foramen magnum meningioma with excessive calcification and no dura tail sign: A case report

**DOI:** 10.1097/MD.0000000000024704

**Published:** 2021-02-12

**Authors:** Li Li, Zhigang Lan, Seidu A. Richard, Yuekang Zhang

**Affiliations:** a Department of Neurosurgery, West China Hospital, Sichuan University; 37 Guo Xue Xiang Road, Chengdu, Sichuan; b Department of Medicine, Princefield University, P. O. Box MA 128, Ho-Volta Region, Ghana, West Africa.

**Keywords:** Cranial nerves, extradural, foramen magnum, intradural, meningioma, surgery

## Abstract

**Rationale:**

Foramen magnum meningiomas are very rare lesions. They frequently originate from the arachnoid cells at the dura matter of the craniocervical junction. Foramen magnum meningiomas are challenging for neurosurgeons because of the complex anatomy of foramen magnum. We present a rare case of FMM with excessive calcification and without the dura tail sign which made the lesion mimic a teratoma.

**Patients concerns:**

A 63 years old woman presented with progressive numbness and hyperesthesia of the shoulders and upper limbs for 2 and half years. She also experienced occasional headaches and dizziness with no nausea, vomiting or fever.

**Diagnoses:**

Computed tomography scan, and magnetic resonance imaging revealed a calcified mass at occipital cistern. The lesion did not show the usual “dura tail sign” which made it mimic a teratoma on magnetic resonance imaging. Histopathology established meningioma.

**Intervention:**

The tumor was completely resected via suboccipital approach.

**Outcomes:**

Two years follow-up revealed no recurrence of the lesion and no neurological deficits.

**Lessons:**

We advocate the use of electromyographic and auditory brainstem responses to monitor the inferior cranial nerves because the tumor often adheres to these nerves.

## 1 Introduction

Foramen magnum meningiomas (FMMs) originates from the arachnoid cells at the dura matter of the craniocervical junction.^[1,2]^ Location wise, FMMs forms about 2.6% of all meningiomas.^[1,3]^ Clinically, presentation of FMMs is nonspecific because of their location as well as their craniospinal extension.^[2]^ Nevertheless, occipital headache as well as upper cervical pain, which is often exacerbated by neck flexion or Valsalva maneuvers are early features of FMMs.^[2]^

Radiologically, computed tomography (CT) scan, CT angiography (CTA), magnetic resonance imaging (MRI), and magnetic resonance angiography (MRA) are the commonly used for the diagnoses FMMs as well as visualize adjacent neurovascular structures.^[2,4]^ Four vessel angiographies are very beneficial in identifying particular anatomy shown in MRA during evaluation. Surgery is the gold-standard treatment option for FMM.^[1,2,5]^ Nevertheless, radiation therapy as an adjunct to surgery or as a primary treatment with radiosurgery has shown promising results in local control of the lesions.^[6,7]^ We present a rare case of FMM with excessive calcification and without the dura tail sign which made the lesion mimic a teratoma.

## 2 Case report

**Figure 1:**
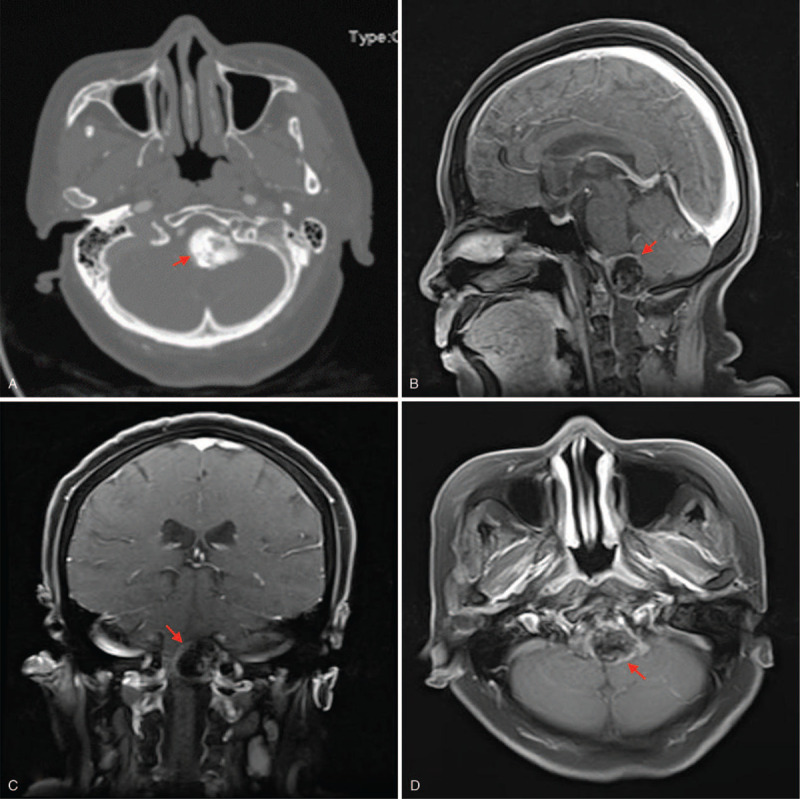
A-D are preoperative CT and MRIs. A: Is a CT scan showing a calcified mass at occipital cistern. B-D: Are MRIs showing an irregular lesion in the posterior part of the medulla oblongata with enhanced edges signifying calcifications. *Red arrow = tumor*. CT = computed tomography, MRI = magnetic resonance imaging.

A 63 years old woman presented with progressive numbness and hyperesthesia of the shoulders and upper limbs for two and half years. She also experienced occasional headaches and dizziness with no nausea, vomiting or fever. Her past medical history was unremarkable. Cranial nerve (CN) examinations revealed deficits in the lower CN (CN IX, X, XI, and XII). The muscle strength in the upper limbs were 4/5 in both limbs with normal reflexes. General physical examination did not yield much. Routine laboratory investigations were grossly at normal rangers. Routine Chest-X ray and electrocardiogram (ECG) were essentially normal. CT scan revealed a calcified mass measuring about 4.1 x 3.5 mm at occipital cistern (Fig. 1 A). All other structures were grossly normal. CTA showed dilated blood vessel around the lesion in the occipital cistern. Also, MRI revealed an asymmetrical lesion in the posterior part of the medulla oblongata measuring about 3.4 x 2.3 x 3.2 cm with enhanced edges signifying calcifications (Fig. 1 B-D). Initial diagnosis was meningioma with calcifications based on the MRI findings although it mimics a teratoma because of the no dura tail sign. Thus, we decided to resect the tumor.

**Figure 2:**
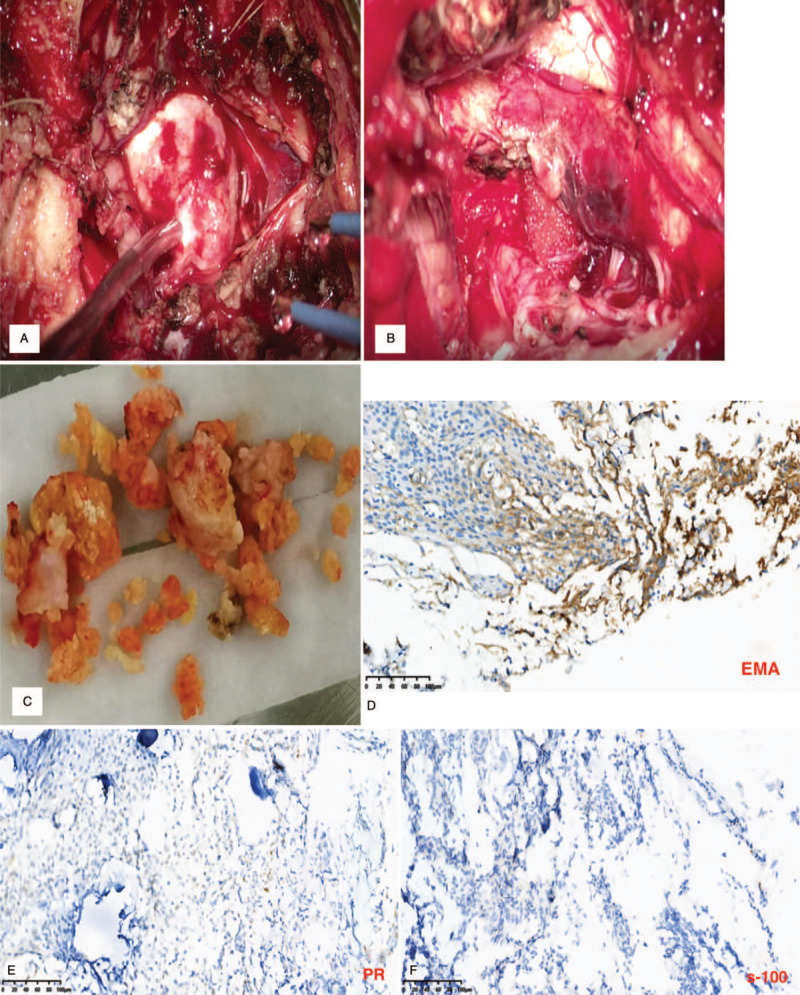
A-F: are intraoperative, resected tumor and histopathology images. A: Is an intraoperative image showing the tumor. B: Is an intraoperative image showing total resection of the tumor. C: Is an image showing the resected tumor. D: Epithelial membrane antigen (EMA) positive stained histopathology image. E: Progesterone receptor (PR) positive stained histopathology image. F: s-100 positive stained histopathology image.

After the general anesthesia, she was also put on the park bench position with her head fixed in Mayfield 3 keys head support system. Electromyographic (EMG) and auditory brainstem responses (ABRs) were utilized to monitor the CN. The tumor was completely resected via suboccipital approach. Laminectomy of the C1 vertebral was also performed to gain further expose of the tumor. Intraoperatively, the tumor was found at the dorsal side of the brain stem in the foramen magnum of the occipital bone. The tumor was solid and soft with areas of calcification as well as very rich blood supply. The tumor was also adhering to inferior CN, posterior inferior cerebellar artery as well as the brain stem (Fig. 2A). After careful dissection, we achieved complete resection of the tumor in a piece meal approach (Fig. 2 B, C). After securing total hemostasis, the occipital bone flap was replaced and the skin closed in layers.

**Figure 3:**
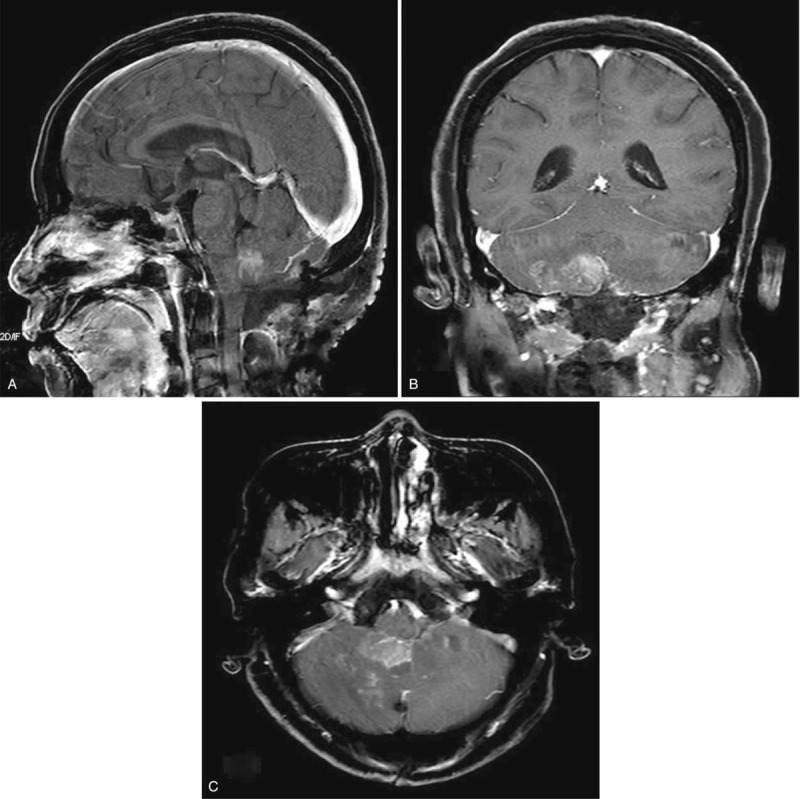
A-C: are postoperative MRIs showing total resection of the tumor. MRI = magnetic resonance imaging.

Examination of the samples at the pathology department revealed the tumor was positive for epithelial membrane antigen (EMA), S-100 and progesterone receptor (PR) which was consistent with diagnosis of meningioma (Fig. 2 D-F). The tumor was classified into World health organization (WHO) Grade-1. Postoperative MRI revealed total resection of the tumor (Fig. 3 A-C). Her postoperative cause was uneventful with no neurological deficits and the patient was discharged home a week after the operation. Two years follow-up revealed no recurrence of the lesion and no neurological deficits.

## 3 Discussion

Meningiomas originates from the arachnoid cells at the dura matter.^[1,3,8]^ The incidence of meningiomas increases with advance age and are most likely to occur in persons older than 60 years of age.^[3,8]^ Meningiomas are more frequent in females than males.^[2,8]^ Our patient was a female in the early sixties.’^[2,8]^ FMMs originates from the craniocervical junction.^[1]^ Based on compartment of development, FMMs are categorized into intradural, extradural, as well as intra- and extradural meningiomas.^[1]^ Furthermore, intradural FMMs are divided into anterior, lateral, and posterior compartments according to dural attachment.^[1]^ Intraoperatively we noticed the lesion had extradural origin which could explain why we did not observe the usual “dura tail sign” on radiology during our evaluation.

Anatomically, the borders of foramen magnum extend from the lower third of the clivus, to upper border of the body of C2 vertebral anteriorly, the jugular tubercle to the upper border of the laminae C2 vertebral laterally, and the anterior edge of the squamous occipital bone to the C2 vertebral spinous process posteriorly.^[2,9,10]^ It houses neural structures like the cerebellar tonsils, inferior vermis, fourth ventricle, caudal part of the medulla, lower CN (CN IX, X, XI, and XII), rostral part of the spinal cord, and upper cervical nerves (C1 as well as C2 vertebrae).^[2,9]^ Furthermore, the vertebral arteries, posterior inferior cerebellar artery, anterior as well as posterior spinal arteries, and the meningeal branches of the vertebral, external, as well as internal carotid arteries are the major arterial structures located within the foramen magnum.^[2,9,10]^ Thus, FMMs are challenging for neurosurgeons because of the complex anatomy above.^[1]^

Initially, FMM presents with occipital as well as posterior cervical pain with or without hyperesthesia in the C2 dermatome.^[1]^ The pain associated with FMMs is unilateral and often aggravated by extension or lateral flexion of the neck, straining, coughing, or sneezing. Some patients present with the “cold clumsy hand” which is the decreased ability to write or button clothes.^[1]^ The classic foramen magnum syndrome often starts with a unilateral arm sensory as well as motor deficits, which advance to the ipsilateral leg, then the contralateral leg, and ultimately contralateral upper extremity.^[2]^ Spastic quadriparesis as well as lower CN palsies are aggravated and serious final symptomatology.^[2]^ Our patient presented with a progressive numbness of both upper limbs accompanied with hyperesthesia on the shoulders. She also experienced occasional headaches and dizziness with no nausea, vomiting or fever. Multiple sclerosis, amyotrophic lateral sclerosis, syringomyelia, as well as cervical spondylosis are the clinical differential diagnosis.^[2,11–13]^

Careful radiological evaluation is very crucial in the diagnosis as well as management of FMMs. CT scan is capable of identifying calcification, hyperostosis, as well as osseous anatomy.^[1,2]^ Furthermore, the extent of bone resection required to resect tumor safely is determined by axial CT scan because of the sharp contrast between bone as well as soft tissues.^[2]^ In our case, CT scan detected a calcified mass at occipital cistern which was consistent with meningioma. CTA also aided in assessing the vasculature of the lesion in the occipital cistern. MRI is the gold standard radiological modality for the evaluation of tumors of FMMs because it offers high-resolution images of soft-tissue anatomy that is not receptive to degradation by the adjacent skull base, a pitfall of CT scan.^[2]^ Plain T1-weighted MR images offer little difference between the tumor and the brainstem because the tumor may appear isointense, mildly hypointense, or hyperintense to adjacent brain.^[2]^

Also, meningiomas appear as isointense to slightly hyperintense compared with brain on T2-weighted images.^[2]^ Furthermore, T2-weighted sequences is capable of detecting edema within the neuroparenchyma which indicates that the pial membrane has been invaded.^[2,14]^ Edema often suggest function preservation with a near-total resection leaving a small thin plating of tumor intact.^[2,14]^ In our case, MRI was capable of detecting a calcified lesion which was consistent with diagnosis of FMM although it mimics a teratoma because of the no dura tail sign. MRA is also valuable because it aids in visualization of vascular structures such as vertebral as well as basilar arteries together with their branches and perforators around the tumor.^[1]^ Intraoperatively, neuro-navigation using MRI or CT images aids in attaining total resection of the tumor as well as locate vertebral arteries around the tumor.^[1]^

The gold-standard therapy for FMMs is surgery.^[2,5,15–17]^ Surgical resection is advocated to alleviate neurological symptomatology and gross total resection is often beneficial.^[4,6,15]^ The classic approach to FMMs is the suboccipital craniotomy, or craniectomy, with or without cervical laminectomy.^[2,17]^ We used the suboccipital approach to assess the tumor and completely resected it. Laminectomy of the C1 vertebral was also performed to gain further expose of the tumor. The tumor was found at the dorsal side of the brain stem in the foramen magnum of the occipital bone intraoperatively. We advocate the use of EMG and ABRs to monitor the inferior CN because the tumor often adheres to these nerves.

Also, the transcondylar approach was developed in an attempt to achieve effective as well as safer resections of lesions situated anteriorly.^[2,16,18]^ Resection of some or all of the occipital condyle is often required in the transcondylar approach.^[2,16]^ Furthermore, the far-lateral approach, which requires removal of the foramen magnum rim towards the condyle as well as excision of the ipsilateral atlantal arch have also been used with success in patients with FMMs.^[19,20]^ On the other hand, radiation therapy has proven to be effective in controlling local lesions when used either as an adjunct to surgery or as a primary therapy.^[6]^ Several radiosurgery sequences have exhibited local control rates of 92% to 100% at 5 years as well as 88% to 95% at 10 years.^[6,7,21]^

Spherical growths of meningothelial cells (whorls) which subsequently mineralize into psammoma bodies is the hallmark of meningiomas.^[8]^ Intranuclear cytoplasmic pseudo-inclusions often referred to as cytoplasmic invaginations in the nuclei as well as central chromatin clearing are often seen in the tumor nuclei.^[8]^ EMA

is the most generally used immunohistochemical marker to detect a meningioma.^[8]^ Nevertheless, the somatostatin receptor 2A has proven to more a superior immunostain target.^[6,22]^ Ki-67 labeling index is often used to quantify proliferative cells within a tumor while MIB-1 monoclonal antibody is used to stain the Ki-67 antigen.^[23,24]^

Glial fibrillary acidic protein positive cells have been associated with invasive meningiomas that are anchored to blood vessels although negative in none invasive meningioma.^[23,24]^ S-100 protein is often advantageous in differentiating meningiomas from schwannomas though 90% of fibrous meningiomas typically express S-100 protein.^[23,24]^ Roser et al found a correlation between high vascularity and positive PR status.^[25]^ EMA, S-100 as well as PR were positive in our histopathological examination which was consistent with diagnosis of a meningioma.

WHO classified meningiomas into Grade 1, 2 and 3.^[26–28]^ Grade-1 designate a slow growth rate without brain invasion, permitting for gross total resection of the tumor and its dura or bone extensions.^[26]^ Grade-2 designates a medium growth rate or brain invasion, and grade-3 indicates a malignant or cancerous tumor.^[26]^ The tumor in our patient was WHO Grade-I.

## 4 Conclusions

We did not observe the usual “dura tail sign” on radiology during our evaluation possible because the lesion had extradural origin. FMMs are challenging for neurosurgeons because of the complex anatomy of foramen magnum. Thus, we advocate the use of EMG and ABRs to monitor the inferior CN because the tumor often adheres to these nerves.

## Author contributions

**Conceptualization:** Li Li, Zhigang Lan, Seidu A. Richard, Yuekang Zhang.

**Data curation:** Li Li, Zhigang Lan, Seidu A. Richard, Yuekang Zhang.

**Formal analysis:** Li Li, Seidu A. Richard, Yuekang Zhang.

**Funding acquisition:** Yuekang Zhang.

**Investigation:** Li Li, Zhigang Lan, Seidu A. Richard.

**Methodology:** Li Li, Zhigang Lan, Seidu A. Richard, Yuekang Zhang.

**Resources:** Zhigang Lan, Seidu A. Richard, Yuekang Zhang.

**Supervision:** Zhigang Lan, Seidu A. Richard, Yuekang Zhang.

**Writing – original draft:** Seidu A. Richard.

**Writing – review & editing:** Li Li, Zhigang Lan, Seidu A. Richard, Yuekang Zhang.

## Abbreviations

Abbreviations: ABRs = auditory brainstem responses, CN = cranial nerve, CT = computed tomography, CTA = computed tomography angiography, EMA = epithelial membrane antigen, EMG = electromyographic, FMMs = Foramen magnum meningiomas, MRA = magnetic resonance angiography, MRI = magnetic resonance imaging, PR = progesterone receptor.
